# Coexisting elastosis perforans serpiginosa and acquired cutis laxa following long-term penicillamine in Wilson disease

**DOI:** 10.1093/skinhd/vzaf060

**Published:** 2025-09-05

**Authors:** Tu-An Ma, Nicholas Van Rooij

**Affiliations:** Department of Dermatology, Princess Alexandra Hospital, Brisbane, QLD, Australia; Department of Dermatology, Princess Alexandra Hospital, Brisbane, QLD, Australia

## Abstract

We report a rare case of coexisting elastosis perforans serpiginosa and acquired cutis laxa in a patient with Wilson’s disease following prolonged penicillamine therapy. This represents two rare but important dermatologic complications of long-term chelation therapy that clinicians should be aware of, given the potential for underlying elastin disruption to also affect systemic tissues.

## Teaser

We describe a rare case of co-existing elastosis perforans serpiginosa and cutis laxa following long-term penicillamine treatment in a patient with Wilson's disease.

**What is already known about this topic?**

- Long-term penicillamine therapy has the potential to cause elastin-related dermatoses.
- These dermatoses may persist for years or may regress slowly.

**What does this study add?**

- Elastin fibre changes may occur extracutaneously, involving vessel walls, lungs and joint capsules; as such, early recognition of these dermatoses in patients on long-term penicillamine is crucial.
- Elastosis perforans serpiginosa and acquired cutis laxa usually occur separately; the coexistence of both secondary to long-term penicillamine is very rare.

## Case report

Elastosis perforans serpiginosa (EPS) and acquired cutis laxa are rare elastin-related dermatoses associated with long-term penicillamine therapy. However, the simultaneous existence of both is exceptionally rare. We describe a case of a patient with coexisting and widespread EPS and acquired cutis laxa, highlighting an unusual dermatological manifestation of long-term chelation therapy, with possible systemic consequences.

**Figure 1:**
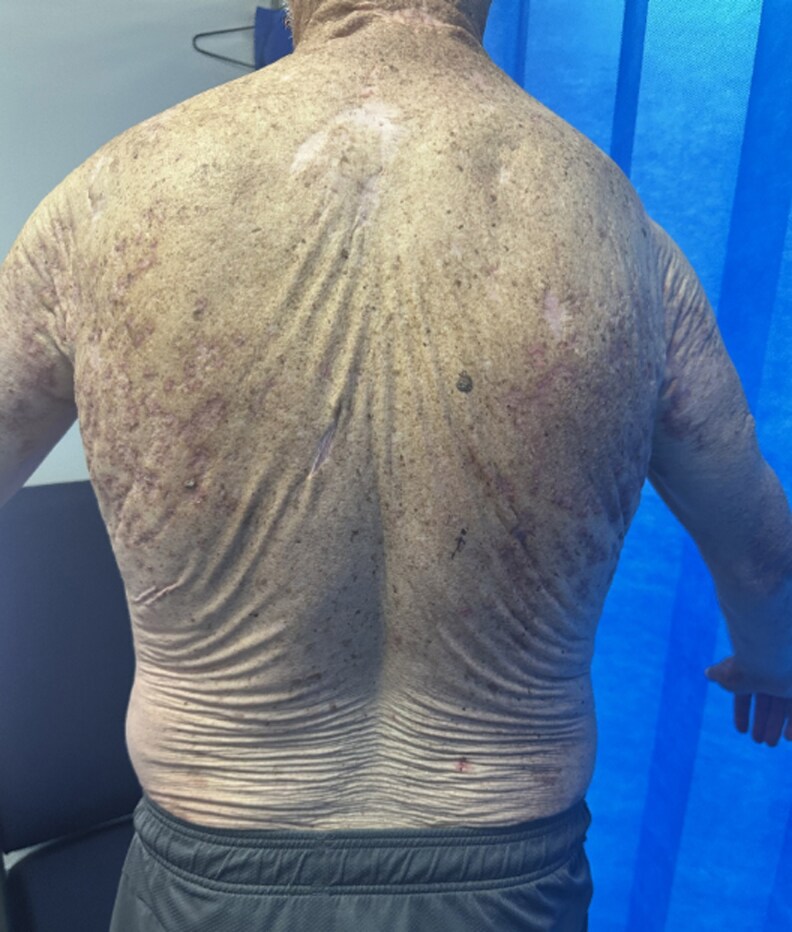
Increased skin laxity to the mid- and lower back with prominent rugose resting skin tension lines.

**Figure 2:**
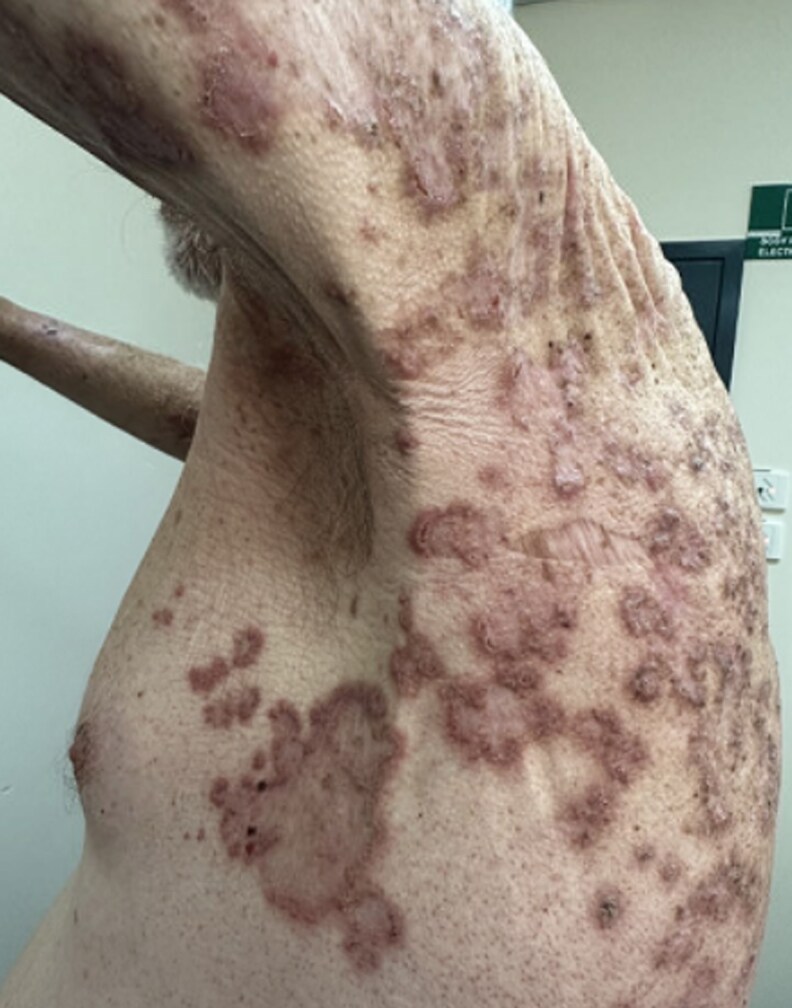
Hyperkeratotic annular and serpiginous plaques with prominent lax skin fold best appreciated in flexural areas, including left axillae.

A 59-year-old man with Wilson disease, who previously received penicillamine for 30 years, presented with an 8-year history of progressive, pruritic, annular plaques predominantly to the trunk and flexures of the proximal limbs, with associated hyperkeratosis and skin redundancy (Figures 1, 2). There were no ophthalmic, oral or joint involvement. Despite prior treatments with topical emollients, antifungals, retinoids and propylene glycol, the eruption progressed. He had discontinued penicillamine 4 years prior to presentation due to neurological side effects and had subsequently changed to oral zinc replacement as chelation therapy. His dermatological history included melanoma *in situ*, which was excised with no recurrence, and multiple keratinocyte cancers.

**Figure 3:**
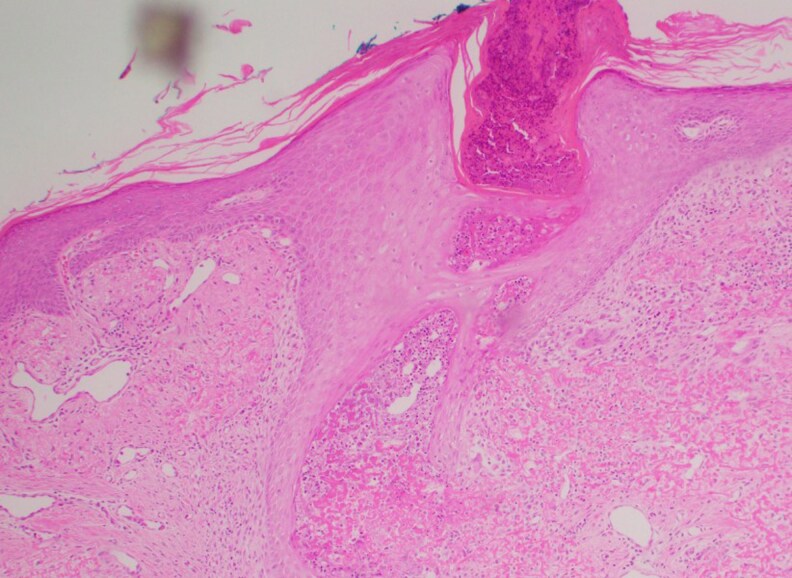
Left upper back punch biopsy stained with haematoxylin and eosin at ×100 magnification. The staining shows multiple foci with transepidermal elimination of basophilic debris, neutrophils and brightly eosinophilic collagen fibres, associated with hyperplastic squamous epithelium. These findings are consistent with elastosis perforans serpiginosa.

**Figure 4:**
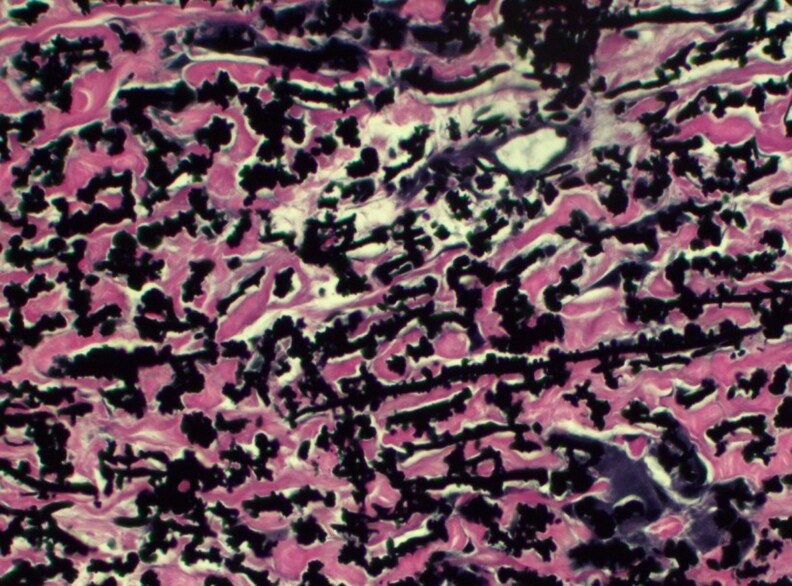
Verhoeff-Van Gieson staining confirming elastosis perforans serpiginosa with typical appearance of thickened, serrated elastic fibres in dense clumps (× 400 magnification).

Punch biopsy revealed hyperplastic epithelium with trans­epidermal elimination of basophilic debris, neutrophils and brightly eosinophilic collagen fibres (Figure 3). Verhoeff-Van Gieson staining demonstrated thickened, serrated elastin fibres, confirming EPS (Figure 4).

His presentation and findings supported coexisting diagnoses of EPS and acquired cutis laxa, likely secondary to prolonged penicillamine. Throughout 5 years of follow-up for skin cancer surveillance, the EPS plaques partially faded with no new lesions. As the pruritus improved modestly and the lesions were not bothersome, the patient declined further treatments.

EPS and acquired cutis laxa are rare dermatoses associated with elastin fibre changes, often induced by penicillamine, a drug used to treat Wilson disease, cystinuria and rheumatoid arthritis.^1^ Up to 50% of patients using penicillamine experience cutaneous adverse effects.^1^ Long-term use induces elastin fibre changes through two primary mechanisms. Firstly, by chelating copper, an essential cofactor for lysyl oxidase, it causes accumulation of abnormal elastin fibres. Secondly, by binding to collagen aldehydes, it inhibits collagen cross-linking.^2,3^ The coexistence of EPS and cutis laxa induced by penacillamine is rarely reported, with reported cases presenting as localized disease rather than widespread involvement.^2,3^

Drug-induced EPS is most commonly associated with penicillamine.^2^ EPS primarily affects young adults, with a male predominance.^3^ It typically presents as progressive annular or serpiginous hyperkeratotic plaques localized to intertriginous areas including the antecubital fossa, axilla and lateral neck folds, but it has also been reported in the lips, oral mucosa and glans penis.^3^ EPS may be misdiagnosed as other annual eruptions including tinea corporis or granuloma annulare. Histopathology demonstrates epidermal hyperplasia, thickened ‘bramble brush’ elastin fibres, transepidermal elimination of nuclear and granular basophilic debris, and sparse mononuclear inflammatory infiltrates.^3^

Acquired cutis laxa is commonly associated with drug exposure to penicillamine or isoniazid, hypersensitivity reactions and inflammatory diseases including systemic lupus erythematosus and syphilis. It is characterized by localized or widespread distributions of loose, pendulous skin. Histopathology demonstrates sparse, fragmented elastin fibres.^4^

There is some evidence to suggest that penicillamine may also induce elastotic changes in vessel walls, lungs and joint capsules.^1,2^ While EPS may regress following drug discontinuation,^2^ it may also progress or remain unchanged, as demonstrated in our case. There is no standardized management for EPS, although commonly trialled treatments include topical retinoids and corticosteroids, cryotherapy, narrow-band ultraviolet B radiation and carbon dioxide or pulsed dye lasers.^2,3^ However, the efficacy of such treatments remains largely anecdotal.^2^

## Data Availability

No new data were generated or analysed in support of this research. Existing data can be found using references. Patient data underlying this article can be shared on reasonable request to the corresponding author.
